# Simultaneous Ship Detection and Orientation Estimation in SAR Images Based on Attention Module and Angle Regression

**DOI:** 10.3390/s18092851

**Published:** 2018-08-29

**Authors:** Jizhou Wang, Changhua Lu, Weiwei Jiang

**Affiliations:** 1 Department of Computer Science and Information Technology, Hefei University of Technology, Hefei 230000, China; jsdzlch@hfut.edu.cn (C.L.); jiangww@hfut.edu.cn (W.J.); 2 Department of Electronic Information Technology and Electric Engineering, Hefei University, Hefei 230000, China

**Keywords:** SAR image, ship detection, angle estimation, attention, deep learning

## Abstract

Ship detection and angle estimation in SAR images play an important role in marine surveillance. Previous works have detected ships first and estimated their orientations second. This is time-consuming and tedious. In order to solve the problems above, we attempt to combine these two tasks using a convolutional neural network so that ships may be detected and their orientations estimated simultaneously. The proposed method is based on the original SSD (Single Shot Detector), but using a rotatable bounding box. This method can learn and predict the class, location, and angle information of ships using only one forward computation. The generated oriented bounding box is much tighter than the traditional bounding box and is robust to background disturbances. We develop a semantic aggregation method which fuses features in a top-down way. This method can provide abundant location and semantic information, which is helpful for classification and location. We adopt the attention module for the six prediction layers. It can adaptively select meaningful features and neglect weak ones. This is helpful for detecting small ships. Multi-orientation anchors are designed with different sizes, aspect ratios, and orientations. These can consider both speed and accuracy. Angular regression is embedded into the existing bounding box regression module, and thus the angle prediction is output with the position and score, without requiring too many extra computations. The loss function with angular regression is used for optimizing the model. AAP (average angle precision) is used for evaluating the performance. The experiments on the dataset demonstrate the effectiveness of our method.

## 1. Introduction

Synthetic aperture radar (SAR) is active radar that can provide high resolution images under all weather conditions. SAR images have been widely used for fishing vessel detection, ship traffic monitoring, and immigration control [1,2]. Numerous studies have been performed to detect ships in SAR images [3,4,5,6,7]. The ship detection methods used in SAR images are usually inherited from the optical remote sensing domain. The detection methods can be divided into four types [8,9,10]: (1) template matching-based object detection methods; (2) knowledge-based object detection methods; (3) object-based image analysis (OBIA)-based object detection methods; and (4) machine learning-based object detection methods. Machine learning-based methods usually have better performance compared with the other three detectors. They extract the candidate object features, such as the histogram of oriented gradients (HOG) [11], scale-invariant feature transform (SIFT) [12], and bag-of-words (BoW) [13], followed by a particular classifier, for example, a sparse representation classifier (SRC) [14] or a support vector machine (SVM) [15].

For a single-channel SAR image, ship detection algorithms generally utilize the amplitude information of the SAR image to discriminate ships from sea clutter. Ship-wake detection is also studied in SAR images [16,17,18]. Many algorithms for ship detection in SAR images, among which are constant false alarm rate (CFAR) and its variations, are widely used [19,20]. They can automatically adapt the threshold to the varying sea background while maintaining the expected performance.

Due to the strong representational power of convolutional neural networks (CNNs) [21,22,23,24,25,26], computer vision tasks such as classification, segmentation, and object detection have been dominated by them. The deep-learning-based object detector can be divided into two types. The first is two-stage method. R-CNN (region-based convolutional neural network) [27] is the first detector that is based on the CNN, and it has impressive performance compared with the old pipelines. It generates region proposals first by some heuristic methods, for example selective search (SS) [28]. Then, every proposal is fed into a CNN for extracting features. However, R-CNN is time-consuming and needs a large amount of memory to store the features. Fast R-CNN [29] improves R-CNN by the region-of-interest (RoI) pooling layer and feature projection. The RoI pooling layer allows the proposals to have different sizes. Feature projection makes all the proposals share computation. Fast R-CNN only computes the whole image once, which reduces the computation significantly. This makes the proposal generation time the bottleneck. Faster R-CNN [30] introduces a region proposal network (RPN) to replace the special region proposal method. The RPN shares convolution computation with the detection network and improves the quality of proposals.

The second type is the one-stage method. YOLO [31] computes a global feature map and uses a fully-connected layer to predict detections in a fixed set of regions. Compared with region-based methods, YOLO no longer requires a second per-region classification operation, making it extremely fast. The single shot detector (SSD) [32] also performs convolutional operations on the entire input image, but produces a fixed-size collection of bounding boxes based on a small set of default boxes at each location in several feature maps. The role of default boxes without computation in the SSD is the same as the RPN in Faster R-CNN.

A deep-learning-based object detector is end-to-end and data-driven, which means it requires as little intervention as possible in the process, and as little as possible to make a hypothesis. We just need to give abundant data to train the models. When performing detection tasks, the trained model just needs an input image to output the result, without any middle pipelines.

In general, the two-stage detector has a higher accuracy and a heavier computational cost. The one-stage detector is faster and simpler to train in the end-to-end way, especially the widely used SSD detector. Also, the accuracy of the SSD is comparable with the two-stage method combined with some innovations [33]. Therefore, we use the SSD as the detector in our paper.

Ship detection methods in SAR images also inherit the above ideas. For example, Li [34] proposed an improved Faster R-CNN, which shows the excellent performance of deep-learning-based detector. The paper also provides a dataset called the SSDD (SAR ship detection dataset) for training and testing algorithms. Zhao et al. [35] proposed a method through feature recognition with an adaptive background window to detect inshore ships in SAR images. Liang et al. [36] presented an approach via saliency and context information to deal with inshore SAR ship detection. However, these methods of inshore SAR ship detection require post-processing to deal with many false alarms, and so they are not end-to-end [37].

Orientation estimation is also an important task for forecasting the direction of the ships’ navigation. The estimation of orientation is done according to its geometric information, as the length and width of a ship are different in general. In the traditional method, the above tasks are accomplished sequentially. This method has the following two disadvantages. It needs to extract rotation-invariant features, and the process is trivial. It is not learning-based and data-driven, so it is easily interfered with by the surrounding environment.

Inspired by [38,39], we adopt the rotatable bounding box to detect ships and estimate their angles. The angles are also information to be estimated just like the position of the ship. Therefore, the construction of a rotation-invariant feature is not needed, as the detector can still detect the ship no matter how the ship rotates. The key to the architecture is a deep convolutional network, where scores for the presence of an object category, the offset for its location, and the approximate angle are all estimated on a regular grid of locations in the image.

Compared with the traditional bounding box, the rotatable bounding box not only can detect ships, but it also can estimate their orientations simultaneously. The rotatable bounding box is tighter, which is robust to the disturbance of the background pixels. This is also helpful for improving the result of detection and angle estimation [40].

It is shown that the frontend of the SSD is too simple to consider the speed and accuracy of the detector. We focus on alleviating these issues by carefully incorporating recent ideas into the front-end sub-network design. We add the semantic aggregation module to boost the accuracy further, just like in FPNs (Feature Pyramid Networks) [41].

Attention was first used in SENet [42,43]. The squeeze-and-excitation block can adaptively recalibrate channel-wise feature responses by explicitly modeling interdependencies between channels. This is helpful for classification. GRP-DSOD (gated recurrent pyramid-deeply supervised object detector) [44] uses a gate in the prediction layer to adaptively enhance or attenuate supervision at different scales based on the input object size. The gate is similar to the SENet. However, they neglect the spatial attention that is helpful for location. As only part of SAR image contains ship pixels, spatial attention plays an important role in deciding ‘where’ to focus [45]. In order to detect ships with different sizes, we add an attention module to the frontend of the SSD. The attention module can take into consideration the channel and space information of ships in SAR images, as the channel-wise attention is helpful for classification, and spatial attention is helpful for location. Therefore, the integrated module is helpful for detecting ships.

The experiments are based on the SSDD dataset. However, the labels are rotatable bounding boxes. By integrating the angle estimation directly into a very fast object detection pipeline, instead of adding it as a secondary classification stage, the resulting detection and angle estimation system is very fast, processing up to 40 FPS (frames per second) on a GTX 1080.

Our main contributions are summarized as follows:

1. We adopt an end-to-end framework to detect ships and estimate their orientations simultaneously. This method can output the location, category, and orientation in a model, without tedious pipelines.
2. The rotatable bounding box is tighter and contains less pixels of background. Therefore, it is easy to distinguish them from the background, especially near the dock.
3. In order to boost the performance further, we propose a semantic aggregation module, which can add semantic information to every layer in a top-down way.
4. An attention module is used for adaptively selecting meaningful features for classification and location.
5. Angular regression is used for predicting angles without increasing the computational load.

## 2. Simultaneous Detection and Angle Estimation

Ship detection and angle estimation are well-studied problems in SAR imagery. Most of the methods seek to perform them in two stages. The two-stage approach separates the detection and angle estimation. The first stage requires an off-the-shelf detector, such as Faster R-CNN. It can produce the bounding boxes of ships. Then, the cropped regions of ships are fed into another CNN for predicting the angle. This requires re-sampling the image at least three times: once for region proposals, once for detection, and once for angle estimation. Though a two-stage pipeline uses a fast detector in stage one, it will still be slower than performing simultaneous detection and angle estimation. This is because the detected objects must be cropped and then processed by a separate network [46].

**Figure 1:**
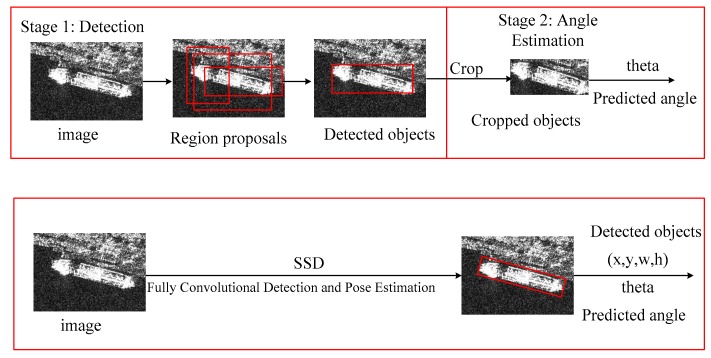
The above is the traditional two-stage method. It detects ships in Stage 1 and estimates their angles in Stage 2. The bottom is the proposed one-stage method. The angle estimation is embedded into the SSD detector, and the category, position, and angle are given through only one propagation.

The one-stage method can integrate the two steps into one, which shows high speed and accuracy, as shown in Figure 1. The traditional pipeline has two stages as shown in the above section of Figure 1. Stage 1 is used for detecting targets and Stage 2 is used for estimating the angle information. They are sequentially executed. However, the method we propose is executed concurrently, as shown in the bottom of Figure 1. The angle information can be predicted with the coordinates of the bounding box. The angle estimation is embedded into the SSD detector, and the category, position, and angle are found through only one propagation. This only slightly increases the computational requirements, but keeps the whole model training in an end-to-end way. Joint training with angular regression can have the ability of synergy. It requires no re-sampling of the image, and instead relies on convolutions for detecting the ship and its angle in a single forward pass. This offers a large speed up, because the image is not re-sampled, and computation for detection and angle estimation are shared.

## 3. Proposed Method

### 3.1. Overall Architecture

**Figure 2:**
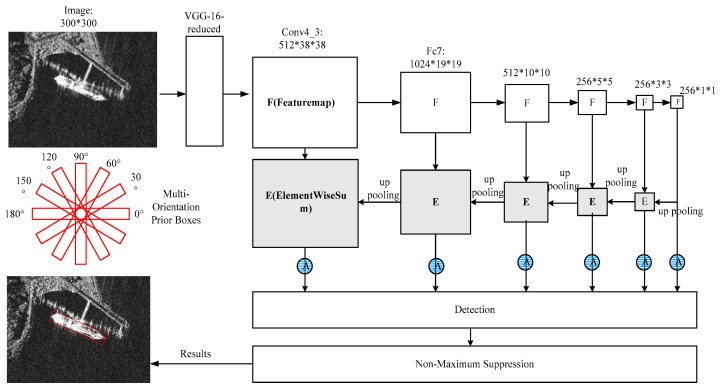
The overall architecture of the proposed method, inherited from the original SSD. It finds ships using rotatable anchor boxes on SAR images and outputs the category, location and orientation of the ships. F represents the feature map, E represents the element-wise summation, and A represents the attention module.

The overall architecture of the proposed method is shown in Figure 2. The backbone architecture is a truncated VGG16 [47]. All the fully connected layers, convolution layers and pooling layers after layer conv4-3 are removed. Then, a 3 × 3 convolution layer is added after layer conv4-3. Our method follows from the original SSD. It finds ships by rotatable anchor boxes on SAR images and outputs the category, location and orientation of each ship. The input SAR images are first resized to 300 × 300 pixels. Then, a truncated VGG16 is used for extracting features. The predictions are done at six different resolutions. They are 38 × 38, 19 × 19, 10 × 10, 5 × 58, 3 × 3, and 1 × 1. These different resolutions can cope with ships with different sizes, especially small ones. Multi-orientation prior boxes (anchor boxes) with different sizes, aspect ratios, and angles are used for generating default boxes. The semantic aggregation module is used for fusing low-level and high-level features in a top-down way. It is helpful for detecting small ships. The attention module is used on every prediction layer for adaptively selecting meaningful features. The whole pipeline is optimized with a multi-task loss function. After the NMS (non-maximum suppression), the position, class, and angle information are output in a forward computation.

The proposed detector has the four differences compared with the original SSD.

The first is the supervised label, which changed from (x, y, w, h, c) to (x, y, w, h, a, c), where x and y denote the top-left coordinate of the bounding box, w and h denote the width and height of the bounding box, c denotes the category of the target, and a denotes the angle of the bounding box.

The second is the semantic aggregation module which includes the up pooling sub-module and the element wise summation sub-module, as illustrated in Figure 2. Low-level features have accurate position information, and high-level features have abundant semantic information. Feature fusion is widely used in object detection tasks [48], as it can detect objects in different sizes. Pyramid is a natural way to realize feature fusion. We apply FPN to the front-end sub-network of the original SSD. FPN exploits the inherent multi-scale, pyramidal hierarchy of deep convolutional networks to construct feature pyramids with marginal extra cost. A top-down architecture with lateral connections is developed for building high-level semantic feature maps at all scales. FPN shows significant improvement as a generic feature extractor in several applications. They can aggregate more semantic information to the former layer, and thus can detect more small objects. An element-wise summation is used to aggregate the features in every stage, as shown in Figure 2. F represents the feature map, E represents the element-wise summation, and A represents the attention module we use in Section 3.2. The up-pooling layer is achieved by nearest neighbor and a 1 × 1 convolution.

The third is the attention module attached to the frontend of the SSD. The attention module includes channel-wise attention and spatial attention. The combination of the two attentions allows the model to take consider classification and location, and thus is helpful for detecting ships.

The fourth is the angular regression module. We use anchors with different sizes, aspect ratios, and orientations. Angle estimation is integrated into the bounding box regression task, and thus, the two tasks can be optimized jointly. This can save a lot of computation, as the prediction only needs a single forward computation.

For an input SAR image, a single computation of the model network is performed and it produces scores for category, bounding box offset, and angle. These are filtered by non-max suppression to produce the final output.

### 3.2. Attention Module

SENet [42] proposes channel-wise attention by the squeeze-and-excitation operation and won the first place in the ILSVRC 2017 classification challenge. SENet is the first attention module used in CNN. The squeeze-and-excitation block can adaptively recalibrate channel-wise feature responses by explicitly modelling interdependencies between channels. It can adaptively select meaningful features for separating ships and non-ships. It is helpful for the task of classification. It is firstly used in object detection task in GRP-DSOD. The gate used in GRP-DSOD only considers the channel attention. Applying a channel attention mechanism in a channel-wise manner can be viewed as a process of selecting semantic attributes. Channel attention can focus on “what” is meaningful given an input image, but in general, a ship only relates to partial regions of an image. A spatial attention mechanism attempts to pay more attention to semantically-related regions. We propose that for ship detection, pixel-wise spatial information is more informative. Space attention focuses on “where” meaningful information is given an input image. Hence, we bring channel and spatial attention simultaneously to the front end.

**Figure 3:**
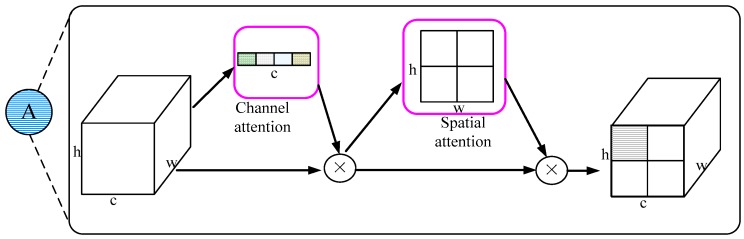
The proposed attention module includes the channel attention and spatial attention.

To achieve this, we sequentially apply channel and spatial attention modules. The attention module in the six prediction layers is shown in Figure 3. The symbols h, w, and c represent the height, width, and the channel of the feature map, respectively. Channel attention can get a vector with c elements, which is used for multiplying the feature map in the height and width dimension. Spatial attention can get a matrix with height h and width w, which is used for multiplying the feature map in the channel dimension. Different colors in Figure 3 represent the different values (weights) of the element.

Each of the branches can learn “what” and “where” to attend in the channel and spatial axes, respectively. As a result, our module efficiently helps the information flow within the network by learning which information to emphasize or suppress.

Given an intermediate feature map $F\in R^{C\times H\times W}$, the attention module generates the channel feature $A_{c}\in R^{C\times 1\times 1}$ and the space feature $A_{s}\in R^{1\times H\times W}$.

(1) $$\begin{array}{l} F^{\prime }=A_{c}(F)\;⊗F\\ F^{\prime \prime }=A_{s}\left(F^{\prime }\right)⊗F^{\prime } \end{array}$$

where ⊗ represents the element-wise multiplication after the broadcasting bt Python.

Channel attention module. Different from the traditional SENet, we use max-pooling to compute channel-wise attention.

(2) $$A_{c}\left(F\right)=\sigma \left(FC\left(MaxPool\left(F\right)\right)\right)$$

where σ represents the sigmoid function and FC represents the three fully connected layers. Compared with the average pooling used in SENet, max-pooling can select the dominant features in the feature map.

Spatial attention module. We generate a spatial attention map by utilizing the inter-spatial relationship of features. Spatial attention focuses on “where” an informative part is, which is helpful for location

(3) $$A_{s}\left(F\right)=\sigma \left(Conv\left(MaxPool\left(F\right)\right)\right)$$

where σ represents the sigmoid function, Conv represents convolutional layer, and MaxPool represents the max-pooling operation.

The combination of the channel attention and spatial attention modules can learn “what” and “where” to look. The former is helpful for classification and the latter is helpful for location. Therefore, the attention module can improve ship detection.

### 3.3. Rotatable Bounding Box

**Figure 4:**
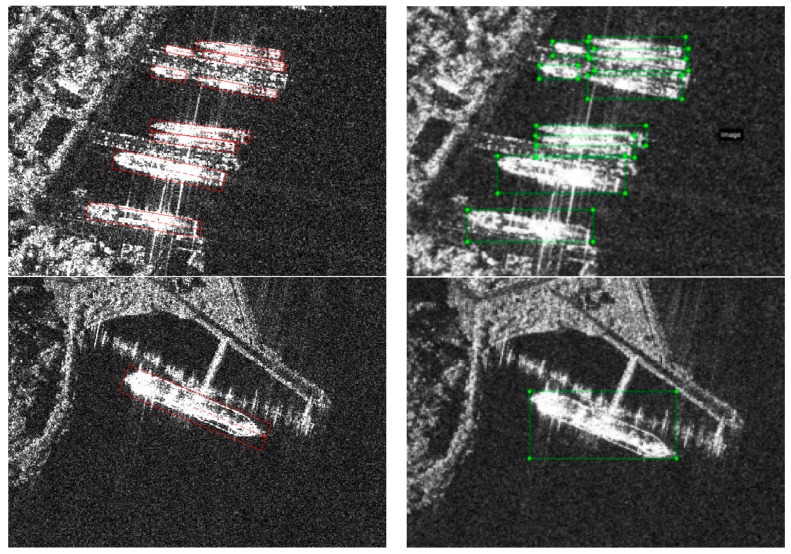
Comparisons between vertical bounding boxes and rotatable bounding boxes.

The traditional method usually detects ships first and then estimates their orientation second. The detection results are usually vertical bounding boxes, but the rotatable bounding box is better for the following reasons. As shown in Figure 4, the red line is the rotatable bounding box and the green line is the corresponding vertical bounding box.

1. The aspect ratio and size of vertical bounding boxes are not identical to the real shape of ships. The width and height of the rotatable bounding box show the real size of ships. Therefore, we can design reasonable prior boxes, as shown in the first row of Figure 4.
2. The vertical bounding box cannot separate the ship and its background pixels in comparison to rotatable bounding boxes. Generally, most of the region inside the vertical bounding box belongs to background pixels. Therefore, it is easy to perform the classification task, as shown in the second row of Figure 4.
3. Rotatable bounding boxes can efficiently separate dense objects with no overlapped areas between nearby targets. Dense objects are difficult to separate. The rotatable bounding box can detect and estimate orientation simultaneously, in a totally end-to-end way, without pipelines, as shown in the first row of Figure 4.

Intersection-over-Union (IoU) is usually used in detectors for selecting positive examples during training. It is also used for NMS (non-maximum supression). The IoU between two boxes is calculated by

(4) $$\mathrm{IoU}(A,B)=\frac{\mathrm{area}(A\cap B)\;}{\mathrm{area}(A\cup B)}$$

where *A* and *B* are bounding boxes without rotation.

Angle-related IoU (ArIoU) is used for a rotatable bounding box detector to perform the same task as above

(5) $${\mathrm{ArIoU}}_{180}(A,B)=\frac{\mathrm{area}(A\cap B)}{\mathrm{area}(B\cup B)}|\cos\left(\theta_{A}-\theta_{B}\right)|$$

where $\theta_{A}$ and $\theta_{B}$ are the angles (in radians) of rotatable bounding boxes *A* and *B*. ArIoU takes angle information into account so that the model can learn to predict the angle of the ship.

IoU and ArIoU are used in different ways. ArIoU is used for training so it can enforce that the detector learns the correct angle, while IoU is used for NMS, so the predictions with inaccurate angles can be effectively removed.

### 3.4. Multi-Orientation Anchors

In the original SSD, conv4-3, conv10-2, and conv11-2 have aspect ratios of 1, 2, and 1/2, respectively, and conv7 (fc7), conv8-2, and conv9-2 have aspect ratios of 1, 2, 1/2, 3, and 1/3, respectively. The predictions are performed on six different resolutions: 38×38 (conv4-3), 19 × 19 (conv7), 10 × 10 (conv8-2), 5 × 5 (conv9-2), 3 × 3 (conv10-2), and 1 × 1 (conv11-2). Therefore, the SSD has 8732 (38 × 38 × 3 + 19 × 19 × 5 + 10 × 10 × 5 + 5 × 5 × 5 + 3 × 3 × 3 + 1 × 1 × 3) anchors.

In practice, we must choose scales and aspect ratios for default boxes to best fit SSDD dataset. In the SSD, the lowest layer has a scale of 0.2 and the highest layer has a scale of 0.9, and all layers between them are regularly spaced, according to the formulation

(6) $$s_{k}=s_{\min}+\frac{s_{\max}-s_{\min}}{m-1}\left(k-1\right),\;k\in \left[1,m\right]$$

In contrast to the large, medium, and small sizes in PASCAL VOC (PASCAL VOC is a dataset usually used for training and testing generic object detection in computer vision) [49], ships in the SSDD are rather small. Therefore, according to the sizes of ships in the SSDD, we set $s_{\max}=0.3$ and $s_{\min}=0.06$, and we change the minimum and maximum aspect ratio from 0.5 and 2 to 0.25 and 4, respectively.

We replace the aspect ratio of 2 with 3 in conv4-3 and conv 7, increase the aspect ratio to 4 in conv8-2, and increase aspect ratio to 4 in conv10-2 and conv11-2, increasing the number of anchors to 9102.

In order to design the multi-orientation anchors, we use another parameter to control the angle of the anchor box. The angles are 0°, 30°, 60°, 90°, 120°, and 150°. If each location of the feature map rotates at the above six angles, this would produce a lot of anchors (9102 × 6). In order to reduce the number of anchors and maintain accuracy, we use the following setting: conv4-3 has the angle of 0 and 60°, conv7 has the angles of 30° and 90°, conv8-2 has the angles of 60° and 120°, conv9-2 has the angles of 90° and 150°, conv10-2 has the angles of 30° and 120°, and conv11-2 has the angles of 60° and 150°. The number of anchors is then 9102 × 2.

### 3.5. Loss Function with Angle Regression

The deep-learning-based object detectors always use bounding box regression to improve their location ability. Bounding box regression was introduced in R-CNN and improved in Fast R-CNN, adopting translation to the center point of the bounding box, and a transform of the height and width in log space. The regression and the classification task combined are jointly optimized. Here, here we also regard the angle estimation problem as a regression task and integrate it into the bounding box regression. The location loss can be reformulated as

(7) $$L_{loc\;}\left(t^{u},v\right)=\sum_{i\in \left\{x,y,w,h,a\right\}}smooth_{L_{1}}\left(t_{i}^{u}-v_{i}\right)$$

Smooth *L*_1_ is formulated as

(8) $$smooth_{L_{1}\;}\left(x\right)=\left\{ \begin{array}{l} 0.5x^{2}\;\;if\left|x\right|<1\\ \left|x\right|-0.5\;otherwise \end{array}\right.$$

The regression can be formulated as

(9) $$t_{x}=\left(\cos\alpha \left(G_{x}-P_{x}\right)+\sin\alpha \left(G_{y}-P_{y}\right)\right)/P_{w}$$

(10) $$t_{y}=\left(-\sin\alpha \left(G_{x}-P_{x}\;\right)+\cos\alpha \left(G_{y}-P_{y}\right)\right)/P_{h}$$

(11) $$t_{w}=\log\left(G_{w}/P_{w}\;\right)$$

(12) $$t_{h}=\log\left(G_{h}/P_{h}\;\right)$$

(13) $$t_{a}=\left(G_{h}-P_{a}\;\right)/\left(\lambda 180\right)$$

where u denotes the class label, $v=\left(v_{x},v_{y},v_{w},v_{h},v_{a}\right)$ is the ground truth of rotatable bounding box regression target, $t^{u}=\left(t_{x}^{u},t_{y}^{u},t_{w}^{u},t_{h}^{u},t_{a}^{u}\right)$ is a predicted tuple for v, $P=\left(P_{x},P_{y},P_{w},P_{h},P_{a}\right)$ is the proposed rotatable bounding box, $G=\left(G_{x},G_{y},G_{w},G_{h},G_{a}\right)$ is the ground-truth rotatable bounding box, and *λ* is a constant number (*λ* = 0.5).

In the test stage, we can transform an input proposal *P* into a predicted ground truth rotatable bounding box $\hat{G}=\left({\hat{G}}_{x},{\hat{G}}_{y},{\hat{G}}_{w},{\hat{G}}_{h},{\hat{G}}_{a}\right)$ by the transformation

(14) $${\hat{G}}_{x}=t_{x}P_{w}\cos\alpha -t_{y}P_{h}\sin\alpha +P_{x}$$

(15) $${\hat{G}}_{y}=t_{y}P_{w}\sin\alpha +t_{y}P_{h}\cos\alpha +P_{y}$$

(16) $${\hat{G}}_{w}=P_{w}\exp\left(t_{w}\right)$$

(17) $${\hat{G}}_{h}=P_{h}\exp\left(t_{h}\right)$$

(18) $${\hat{G}}_{a}=\lambda 180t_{a}+P_{a}$$

The training procedure is similar to the SSD. Before training, each ground truth rotatable bounding box is assigned several prior boxes according to their ArIoUs.

A bounding box is regarded as positive when the ArIoU with the ground truth is >0.5. After the matching, most of the prior boxes are regarded as negatives. A fixed ratio of 1:3 is chosen to keep the balance for converging at a better point.

The overall training lost can be formulated as

(19) $$L(x,c,l,g)=\frac{1}{N}(L_{conf\;}\left(c\right)+L_{loc}\left(x,l,g\right))$$

where *N* is the number of matched prior rotatable bounding boxes. The confidence loss $L_{conf}\left(c\right)$ is a two class Softmax loss over all selected positive and negative samples, where *c* is the two-dimension confidence vector. The confidence loss is the Softmax loss.

(20) $$L_{conf\;}(x,c)=-\sum_{i\in Pos}^{N}x_{ij}^{1}\log\left(d_{i}^{1}\right)-\sum_{i\in Neg}^{N}\log\left(d_{i}^{0}\right)$$

(21) $$d_{i}^{p}=\frac{\exp(c_{i}^{p})\;}{\sum_{p}\exp(c_{i}^{p})}$$

where $x_{ij}^{p}$ is an indicator for matching the *i*-th default box to the *j*-th ground truth box of category *p*.

The newly added angle regression term uses the tangent function to measure the offset and can make the model learn to estimate the angle of the ship.

## 4. Results

### 4.1. Dataset

We use the SSDD with rotatable bounding boxes to train and test the detector. For each of the ships, we predict the bounding box with a confidence score and an angle. The SSDD has similar labels as the PASCAL VOC dataset. We divide the dataset into three parts (training set, test set, and validation set) with the ratio of 7:2:1. Statistics for the number of ships and images in the SSDD are given in Table 1. NoS is the abbreviation of number of ships, NoI is the abbreviation for the number of images. From Table 1, we can see that most of the SAR images in the SSDD have one ship. Furthermore, only a small number of SAR images have greater than eight ships. Therefore, ships are sparse in the dataset.

**Figure 5:**
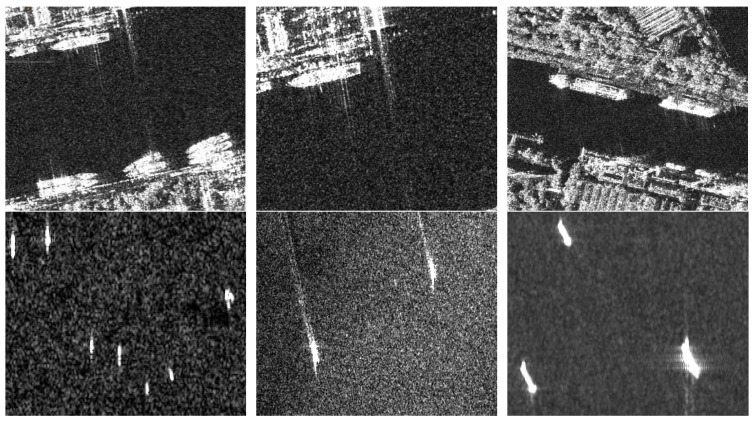

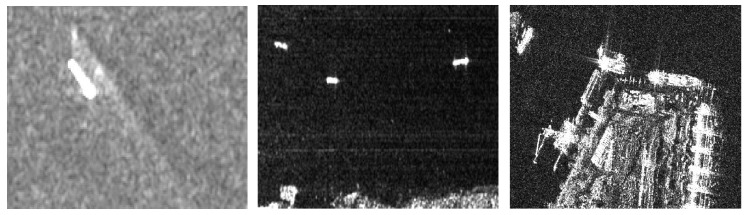
Some examples of SSDD. **The first line** shows ships in complex scenes; **the second line** shows ships in open sea area; **the third line** shows ships with different resolutions.

In the SSDD, there are 1160 images and 2456 ships. The average number of ships per image is 2.12. The dataset is expanded according the demands of the algorithms in the future. Compared with the 9000+ images in the PASCAL VOC dataset with 20 categories, the SSDD is big enough to train a one-class detection model combined with many tricks to prevent over-fitting. As some small ships only have very few pixels in low resolution, sometimes it is hard to decide whether it is a ship or not. If the number of pixels is more than three, we would regard it as a ship and make the annotation. Figure 5 shows some examples from the SSDD. We can see that ships in the SSDD are depicted in a variety of situations. Many ships are near the shore and arranged together, for example the first line of Figure 5, and some ships are in the open sea area, for example the second line of Figure 5, which are easily detected using the traditional method. However, ships near the shore may not be detected by such methods. The third line in Figure 5 shows some ships with different resolutions.

### 4.2. Details

The convolutional architecture, prior anchor box and experiment setup are as follows. We adopt the pre-trained VGG-16 as the backbone network. The backbone is a reduced VGG-16 pre-trained on IMAGENET [50]. All the layers are removed after conv4-3. The extra feature layers are added to detect ships in multi-scale feature maps (38 × 38, 19 × 19, 10 × 10, 5 × 5, 3 × 3, and 1 × 1). In order to detect small ships, we fuse feature maps in a top-down way with semantic aggregation module. As it is hard to distinguish the heads with the tails of ships in SAR images, we only predict the orientation in the range of 0–180 and neglect the direction of navigation.

We train the improved ship detector model on the SSDD. For implementation, we adopt the Caffe [51] framework with Python language to train our deep learning models. The proposed method is evaluated on a 64-bit Ubuntu 14.04 computer with CPU Intel(R) Core(TM) i7-6770K @ 4.00GHz ×8 and NVIDIA GTX1080 GPU with 8G memory CUDA8.0 cuDNN5.0.

We resize the width and height of the images to 300 pixels. If we use batch normalization, the base learning rate is 0.0004. We change the number of classes from 21 to 2. The rotation angles are 0°, 30°, 60°, 90°, 120°, and 150°. The batch size and accumulated batch size are both 16. The maximum iteration is 30 K, the learning policy is multistep with the step value of 10 K and 13 K. The weight decay gamma and momentum value are 0.0005, 0.1, and 0.9 respectively.

The result file contains several lines where each line corresponds to one bounding box. Each line contains seven numbers. The first two numbers are position of the center point of the bounding box. Numbers 3–4 are width and height of the bounding box. The fifth is the label of the object, which is fixed to 1. The sixth number is the angle of this bounding box in degrees. The seventh is the score. The detected objects are sorted by their scores.

### 4.3. Experiments

#### 4.3.1. Evaluation Indicator

AP (average precision) is usually used for evaluating the performance of the detector. AP is calculated as

(22) $$\mathrm{AP}=\int_{0}^{1}P(R)\;dR$$

where *R* represents the recall rate and *P* represents the precision. We can evaluate the accuracy of the predicted angle using the AAP (average angle precision). AAP is an extension of the standard AP metric used to evaluate object detection. In computing AAP, an output from the detector is considered to be correct if and only if the bounding box overlap is larger than 50% and the angle is correct (i.e., the distance between the two angles is smaller than the threshold). FPS is used for evaluating the running time of the detector.

#### 4.3.2. Overall Performance

We compare the two-stage and one-stage methods, and the results are shown in Table 2. For the two-stage method, Faster R-CNN and the SSD are used in the first stage for detection, and AlexNet is used for estimating the orientation of the detected target. From Table 2, we can see that the one-stage method has an obvious advantage compared with the two-stage method. Also, our proposed method is 2.3 percent higher than the DRBox (detection with rotatable boxes) in [39].

Table 3 shows the ablation results of the proposed ideas. We can see that if we move out the semantic aggregation module the AAP is 82.0%, and if we move out the attention module the AAP is 83.7%. If we move out both of them, the AAP is 81.1%. These results show the effect of the two modules.

Table 4 shows the speed comparison of the two-stage and one-stage methods. The two-stage methods have a low FPS compared with the one-stage methods. This is because the second stage needs more computations. However, the extra computation in the second stage can be saved by using a one-stage method. The integrated idea can also give the whole model the function of synergy, which also improves performance. Therefore, we can find that the one-stage method not only has good performance, but also has a fast speed. What should be noticed is that the angle prediction in the two-stage method may include several ships cropped in the first stage. Every cropped ship should feed into the AlexNet for regression.

Table 5 shows the time and accuracy trade-off of the proposed one-stage method. The number 8732 means that the scales and aspect ratios of the anchors we use are the same as the original SSD. The number 9102 means that we replace the aspect ratio of two with three in conv4-3 and conv7, increase the aspect ratio to four in conv8-2, and increase the aspect ratio to four in conv10-2 conv11-2, increasing the number of anchors to 9102. The ×2 and ×6 indicators are also illustrated in Section 3.4. We find that when the number of anchors increases, the performance improves a little, but the computation requirement is considerably raised. Therefore, we adopt the number of anchors of 9102 × 2. In this setting, the AAP is 84.2%, and the FPS is 40.

#### 4.3.3. Detected Ships

**Figure 6:**
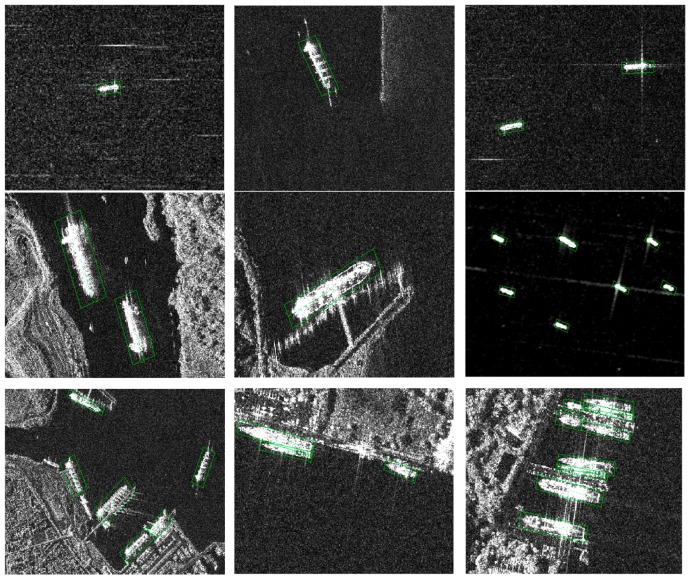
Some detected results in the SSDD. From the results we can see that the proposed method can detect ships with oriented bounding boxes, and thus has the angle information. The method can also detect most of the ships near the shore, which shows impressive performance compared with the traditional detector.

Figure 6 shows some detected results from the SSDD. From the results, we can see that the proposed method can detect ships with oriented bounding boxes, and thus has the angle information. The method can also detect most of the ships near the shore, which shows impressive performance compared with the traditional detector. The CFAR-based detector can only detect ships in the open sea area, and when the image varies greatly they would fail. What is more, it can detect both big and small ships.

**Figure 7:**
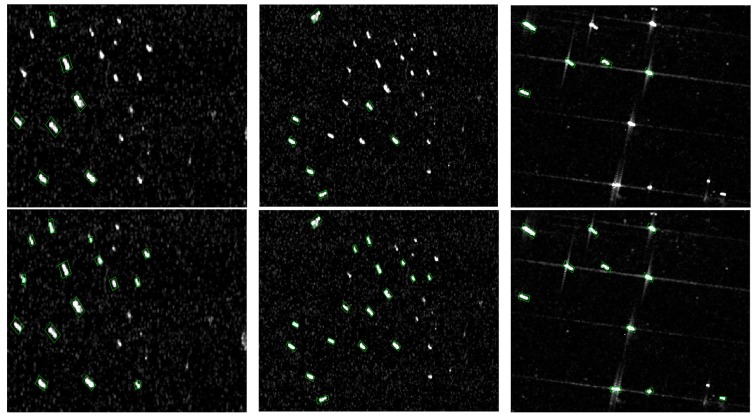
Results of the semantic fusion and attention module. **The top row** shows the detector without the semantic fusion and attention module; **the bottom row** shows the detector with the semantic fusion and attention module.

Figure 7 shows the results of the semantic fusion and attention modules. The first row shows the detector without the semantic fusion and attention modules. The second shows the detector with the semantic fusion and attention modules. We can see that after the two modules, the detector can detect most of the small ships. This is mainly because they can fuse different features and adaptively select meaningful features, which is helpful for those small targets.

### 4.4. False Alarms and Misses

**Figure 8:**
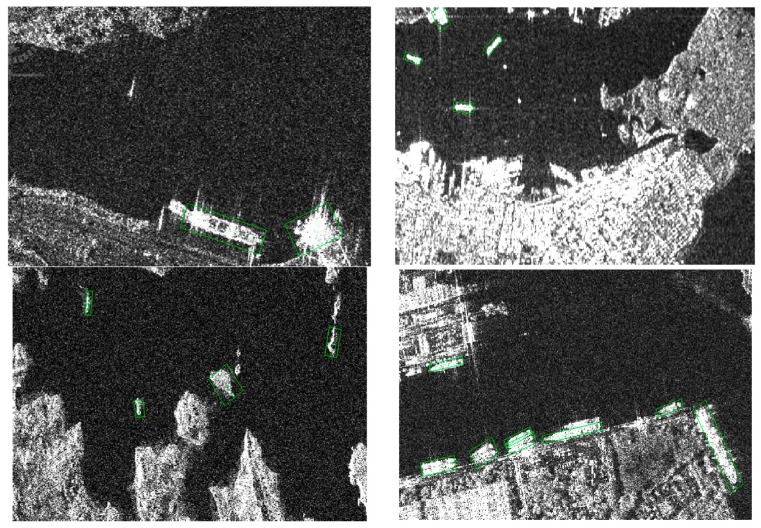
Some false alarms and missed ships in the SSDD. From the above images, we can see that the strong scatter and isolated island are easily detected as ships. Some ships near shore are easily neglected. **Top-left** shows a target in the bottom-right is wrongly detected; **top-right** shows a target in the top-left is wrongly detected; **bottom-left** shows two targets in the left are wrongly detected; **bottom-right** shows the ship is missed in the middle.

The proposed detector is not able to detect all the ships. We select some alarms and missed ships as shown in Figure 8. From the first three images we can see that the detector is easily confused by some strong scatter and small islands. This is mainly because most of the ships have high amplitude, and the strong scatter is similar to a ship.

From the fourth image, we can see that ships near the shore are easily neglected by the detector, especially when densely arranged, when sometimes they are detected as one target.

We randomly choose 200 images to estimate the four probabilities. There are 513 ships in total. We detect 545 ships, among which 480 are correctly detected, 65 are wrongly detected, and 15 are missed, giving a hit probability of 88%. The miss probability is 3% and the false alarm probability is 12%.

## 5. Discussions

The performance of the proposed method relies on the detection results. The improvements are made primarily to the detection performance. We also find that the proposed method can simultaneously detect and estimate the angle in a single forward convolutional neural network computation, with an oriented bounding box which is a better fit with the ships in SAR images. Some small ships and ships in complex backgrounds may also be neglected. This might be solved by increasing the corresponding number of training data.

## 6. Conclusions

Ship detection and orientation estimation is usually a two-stage task. In this paper, we replace the traditional vertical bounding box with the rotatable bounding box, and it can detect and estimate ships simultaneously in only one stage. The rotatable bounding box is much tighter and is better for the classification subtask. We also add the semantic aggregation module to boost the performance, and the attention module is also used to adaptively select meaningful features and neglect weak ones. The multi-orientation anchors are used to generate proposals with multiple scales, aspect ratios and angles. We regard the angle estimation problem as a regression problem, and we embed it into the existing bounding box regression task. This will not increase the computational requirements too much. We demonstrate the result of the proposed method on the SSDD. We find that the proposed method can detect ships and estimate their angles with a high accuracy and speed. What is more, the detector is also more robust and flexible. It can detect ships not only in open sea areas but also near the shore.

## Figures and Tables

**Table 1:** Corresponding relationships between NoS and NoI in SSDD.

NoS	1	2	3	4	5	6	7	8	9	10	11	12	13	14
NoI	725	183	89	47	45	16	15	8	4	11	5	3	3	0

**Table 2:** Accuracy comparison between the two-stage and one-stage pipelines.

Method		AAP (%)
Two-stage	Faster R-CNN + AlexNet	77.8
	SSD + AlexNet	78.5
One-stage	DRBox	81.9
	Proposed	84.2

**Table 3:** Ablation results of the proposed ideas.

Semantic Aggregation	Attention	AAP (%)
×	×	81.1
√	×	82.0
×	√	83.7
√	√	84.2

**Table 4:** Speed comparison between the two-stage and one-stage method.

Method		Detector (FPS)	Angle (FPS)	Total (FPS)
Two-stage	Faster R-CNN + AlexNet	7 (Faster R-CNN)	80	6
	SSD + AlexNet	48 (SSD)	80	30
One-stage	Proposed	-	-	40

**Table 5:** Time and accuracy trade-off of the proposed one-stage method.

No. of Anchors	8732 × 6	8732 × 2	9102 × 6	9102 × 2
AAP (%)	80.2	81.7	84.6	84.2
FPS	15	45	14	40
